# De Novo Transcriptome Assembly of Rice Bean (*Vigna umbellata*) and Characterization of WRKY Transcription Factors Response to Aluminum Stress

**DOI:** 10.3390/plants13223170

**Published:** 2024-11-12

**Authors:** Gunasekaran Ariharasutharsan, Manoharan Akilan, Manickam Dhasarathan, Manivel Amaravel, Sankaran Divya, Mariyappan Deivamani, Manickam Sudha, Muthaiyan Pandiyan, Adhimoolam Karthikeyan, Natesan Senthil

**Affiliations:** 1 Department of Genetics and Plant Breeding, Centre for Plant Breeding and Genetics, Tamil Nadu Agricultural University, Coimbatore 641003, India; 2 Department of Biotechnology, Centre of Excellence for Innovation, Agricultural College and Research Institute, Tamil Nadu Agricultural University, Madurai 625105, India; 3 Department of Genetics and Plant Breeding, Anbil Dharmalingam Agricultural College and Research Institute, Tamil Nadu Agricultural University, Trichy 620027, India; 4 Agro Climate Research Centre, Directorate of Crop Management, Tamil Nadu Agricultural University, Coimbatore 641003, India; 5 Centre of Excellence in Millets, Tamil Nadu Agricultural University, Tiruvannamalai 606603, India; 6 Department of Plant Molecular Biology and Bioinformatics, Center for Plant Molecular Biology and Biotechnology, Tamil Nadu Agricultural University, Coimbatore 641003, India; 7 ICAR-Krishi Vigyan Kendra, Tamil Nadu Agricultural University, Dharmapuri 636809, India; 8 Department of Plant Biotechnology, Center for Plant Molecular Biology and Biotechnology, Tamil Nadu Agricultural University, Coimbatore 641003, India; 9 Agricultural College and Research Institute, Tamil Nadu Agricultural University, Eachangkottai, Thanjavur 614902, India; 10 Subtropical Horticulture Research Institute, Jeju National University, Jeju 63243, Republic of Korea; 11 School of Post Graduate Studies, Tamil Nadu Agricultural University, Coimbatore 641003, India

**Keywords:** legumes, secondary metabolites, transcriptomics, aluminum stress, vigna species

## Abstract

Rice bean is an underutilized legume crop cultivated in Asia, and it is a good source of protein, minerals, and essential fatty acids for human consumption. Moreover, the leaves left over after harvesting rice bean seeds contain various biological constituents beneficial to humans and animals. In our study, we performed a de-novo transcriptome assembly of rice bean, characterized the WRKY transcription factors, and studied their response to aluminum stress. A total of 46.6 million clean reads, with a GC value of 43%, were generated via transcriptome sequencing. De novo assembly of the clean reads resulted in 90,933 transcripts and 74,926 unigenes, with minimum and maximum lengths of 301 bp and 24,052 bp, and N50 values of 1801 bp and 1710 bp, respectively. A total of 27,095 and 28,378 unigenes were annotated and subjected to GO and KEGG analyses. Among the unigenes, 15,593, 20,770, and 15,385 unigenes were identified in the domains of biological process, molecular function, and cellular component, respectively. A total of 16,132 unigenes were assigned to 188 pathways, including metabolic pathways (5500) and secondary metabolite biosynthesis (2858). Transcription factor analysis revealed 4860 unigenes from 98 different transcription factor families. For WRKY, a total of 95 unigenes were identified. Further analysis revealed the diverse response of WRKY transcription factors to aluminum stress. Collectively, the results of this study boost genomic resources and provide a baseline for further research on the role of WRKY transcription factors in aluminum tolerance in rice bean.

## 1. Introduction

Rice bean (*Vigna umbellata*), an unsung grain legume and a member of Asiatic *Vigna*, is considered one of the minor food and fodder crops raised by small and marginal farmers in South and Southeast Asia. Usually grown as an annual, rice bean is a short-lived perennial legume with an erect, semi-erect, or twining growth habit. It is predominantly cultivated at altitudes between 700 and 1300 m above sea level. Although it can grow as tall as 200 cm, it usually stands between 30 and 100 cm [1]. The Indo-China region is believed to be the center of domestication of rice bean. Due to its high yielding potential and ability to grow in marginal lands and drought-prone regions, the crop is gaining interest worldwide [2,3]. The crop possesses the tolerance to biotic and abiotic stresses and has a high nutritional profile [4,5,6]. The dried seeds of the rice bean are usually eaten boiled. They are very nutritious and a great addition to cereal-based diets because the protein is high in lysine [7,8,9]. Young, immature pods are used as vegetables, and immature pods and bean sprouts are highly recommended in a nutritious diet. The whole plant is used as forage for livestock [10,11]. The foliage, leaves, green pods, immature seeds, and flowers are consumed by animals. The vegetative stage of the rice bean also yields a reasonable amount of palatable and nutritious fodder [12]. A substantial amount of byproducts, including leaves and stems, are produced after the harvest of rice bean seeds and are currently either processed as industrial waste or used as animal feed or fertilizer [13]. Several phenolic and flavonoid phytochemicals, such as isovitexin, p-coumaric acid, epicatechin, ferulic acid, quercetin, sinapic acid, catechin, and vitexin, have been reported mainly in the seeds and sprouts of rice bean [14,15]. The compounds have a range of biological and therapeutic benefits for both humans and animals, especially their high antioxidant properties and anti-diabetic α-glucosidase inhibition activity. There is no published literature regarding the area coverage, production, productivity, utilization, or marketing of rice bean. Rice bean is widely cultivated in marginal and unused land, water-deficit areas, and soils contaminated with heavy metals. Aluminum, a heavy metal, contaminates soil and negatively impact plant growth. Therefore, aluminum toxicity acts as a one of the limiting factors affecting the quality and yield of rice bean. Therefore, it is necessary to understand the aluminum tolerance mechanism to improve rice bean production.

RNA sequencing-based transcriptomics is an affordable genomics approach that can be used to understand the mechanism of abiotic stress tolerance. This approach is very useful for discovering major genes and their networks and pathways that influence the stress response in plants [16,17,18]. The WRKY transcription factor family, one of the largest in plants, is distinguished by its highly conserved WRKY domains. These domains regulate gene transcription by specifically binding to the W-box sequence in the upstream region of target genes, playing a crucial role in gene regulation. The WRKY domain consists of a 60-amino-acid polypeptide sequence, featuring a short, conserved sequence (WRKYGQK) at the N-terminal and a zinc finger motif at the C-terminal [19]. This unique structure enables WRKY transcription factors to precisely control gene expression, making them vital components of plant gene regulation. WRKY transcription factors are essential for plant responses to both biotic and abiotic stresses [20]. Research has identified several WRKY transcription factors that play major roles in abiotic stress response. *ZmWRKY40*, induced by drought, high salt, high temperature, and ABA, enhances drought tolerance in *Arabidopsis thaliana* when overexpressed [17]. *ZmWRKY58* from maize increases drought and salt stress tolerance in transgenic rice [21]. *OsWRKY45* from rice improves drought tolerance in Arabidopsis, indicating its involvement in stress response signal transduction [22].

*GsWRKY20* from wild soybean enhances drought tolerance in Arabidopsis [23]. *GmWRKY81* from soybean improves aluminum tolerance when overexpressed [24]. However, *OsWRKY63* from rice negatively regulates cold tolerance, with overexpression leading to increased sensitivity and knockout mutants showing enhanced cold tolerance [25]. Although many studies have demonstrated the role of WRKY transcription factors in abiotic stress, including aluminum stress response in various crops, there is still a lack of comprehensive studies focusing on WRKY TFs in the aluminum stress response. In this communication, we report the rice bean transcriptome obtained from high-throughput RNA sequencing. The unique transcripts generated by the de novo assembly of high-quality reads were examined and annotated. In addition, WRKY transcription factors were characterized and studied for their response to aluminum stress.

## 2. Results

### 2.1. Snapshot of Transcriptome Data Sets and De Novo Transcriptome Assembly

**Figure 1:**
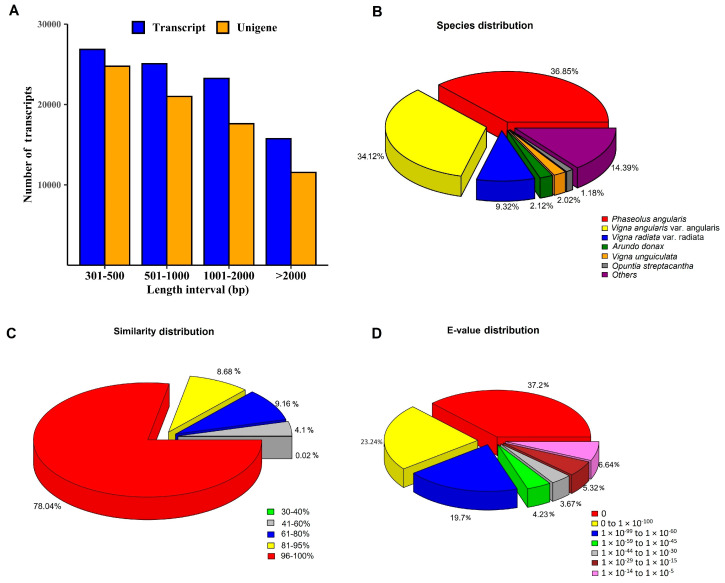
(**A**) Distribution of unigenes and transcript lengths, and summary of annotation statistics from the NR database; (**B**) species distribution; (**C**) similarity distribution; and (**D**) E-value distribution.

The raw data of the rice bean transcriptome were submitted to the NCBI-SRA database and received the accession number PRJNA900023. Adapter sequences and low-quality reads (Phred score < 30) were detached from the raw reads during pre-processing. As a result, 46,144,189 clean reads were obtained, comprising 4.66 Gb of paired-end sequencing data with a GC content of 43% (Table 1). All the filtered high-quality reads were assembled into a de novo assembly using the Trinity assembler. We obtained 90,933 transcripts, with a mean size of 1216.89 bp. These transcripts were grouped into 74,926 unigenes, with a mean length of 1138.24 bp and an N50 value of 1710 bp. A summary of the transcriptome is provided in Table 2. The minimum and maximum lengths of the unigenes were 301 bp and 24,052 bp, respectively (Figure 1A). These results confirm that the unigene integrity is acceptable for downstream analysis.

### 2.2. Functional Annotation of Unigenes

All unigenes were searched using the BLASTx program against the NR, NT, UniProt, KEGG, Pfam, KOG, and GO databases to obtain the comprehensive functional annotations. As presented in Table 3, 74,926 unigenes were annotated by the seven databases. Among the total unigenes, 58,470 (78.03%), 55,350 (73.87%), 56,370 (75.23%), 28,378 (37.87%), 45,905 (61.27%), 49,667 (66.29%), and 27,095 (36.16%) unigenes were annotated to the NR, NT, UniProt, KEGG, Pfam, KOG, and GO databases, respectively. A number of plant species were searched through homologous unigenes, with 36.85% of the annotated unigenes having the maximum resemblance to unigene sequences from common bean (*Phaseolus angularis*), followed by adzuki bean (*Vigna angularis*) (34.12%) and mung bean (*Vigna radiata*) (9.32%) (Figure 1B). From the BLAST results, the percentage of identity values in the 95–100% range was highest at 78.03%, followed by 9.16% in the 60–80% range (Figure 1C). The e-value of 0 recorded a maximum of 37.21%, followed by 23.24% in the 0 to 1 × 10^−100^ range (Figure 1D).

### 2.3. KOG Classification

**Figure 2:**
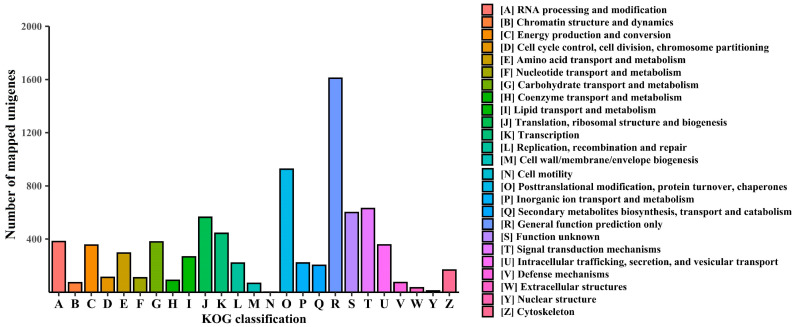
Summary of KOG classification. The X-axis represents the names of the KOG group and the Y-axis represents the number of transcripts under this group in the total annotated genes.

We used the KOG database to explore the functional characteristics of 74,926 unigenes. Of these, 49,667 unigenes were annotated, with 14,327 being distinct KOG-mapped unigenes. The KOG-mapped unigenes were categorized into 25 major groups. The majority of the unigenes (1610) were associated with general function prediction only [R], with another 926, 630, and 600 unigenes associated with [O] posttranslational modification, protein turnover, and chaperones; [T] signal transduction mechanisms; and [S] unknown function, respectively (Figure 2). Furthermore, 13,433 KOG-mapped unigenes were homologous to the species *Arabidopsis thaliana*. A total of 3324 distinct unigenes were aligned with KOG IDs and their corresponding enzyme codes (Supplementary Table S1).

### 2.4. GO and KEGG Analyses

**Figure 3:**
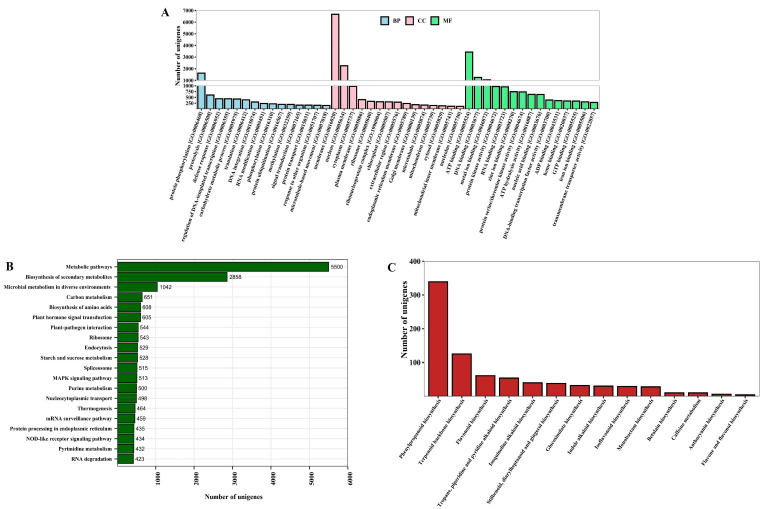
(**A**) Gene ontology classification of unigenes. The histogram shows the results of unigene classification under three major categories of GO terms: biological processes (BP), molecular functions (MF), and cellular components (CC). (**B**) KEGG pathway unigene assignments. (**C**) Details of secondary metabolite biosynthesis.

A total of 74,926 unigenes were subjected to GO analysis. Among them, 27,095 unigenes were annotated with gene ontology. The GO classification includes three major domains: biological process (BP), molecular function (MF), and cellular component (CC). Of these, 15,593, 20,770, and 15,385 unigenes were found in BP, MF, and CC, respectively. The major 15 GO terms are presented in Figure 3A. The prominent terms in the BP domain were defense response [GO: 0006952], carbohydrate metabolic process [GO: 0005975], protein ubiquitination [GO: 0016567], signal transduction [GO: 0007165], and methylation [GO: 0032259]. In the MF domain, notable terms included ATP binding (GO: 0005524), protein kinase activity [GO: 0004672], protein serine/threonine kinase activity [GO: 0004674], DNA-binding transcription factor activity [GO: 0003700], and GTP binding [GO: 0005525]. For the CC domain, the main terms were cytoplasm [GO: 0005737], chloroplast [GO: 0009507], endoplasmic reticulum membrane [GO: 0005789], Golgi membrane [GO: 0000139], and mitochondrion [GO: 0005739]. Further, KEGG pathway enrichment analysis was used to provide functional classification and pathway assignment for 28,378 KEGG annotated unigenes, and a total of 18,081 unigenes were assigned to various pathways. Most of the identified transcripts were mapped to the metabolic pathways (5500), while other major pathways included biosynthesis of secondary metabolites (2858), microbial metabolism in diverse environments (1042), plant hormone signal transduction (605), plant–pathogen interaction (544), starch and sucrose metabolism (528), MAPK signaling pathway (513), purine metabolism (500), and pyrimidine metabolism (432). The KEGG pathway analysis results are presented in Figure 3B.

### 2.5. Summary of SSRs

A total of 6711 SSR loci were identified from 74,926 unigene sequences of rice bean, and 5700 of these sequences contained SSR. Among the repeats, di-nucleotide repeats were most abundant (52.53%), followed by tri-repeats (43.63%), tetra-repeats (2.89%), penta-repeats (0.43%), and hexa-repeats (0.52%) (Table 4). Among the di-repeats, the AG-type repeat was the most frequent, accounting for 18.89%, followed by the TC repeat (17.39%). The least frequent di-repeats were the CG repeat (0.11%) and GC repeat (0.09%) (Supplementary Table S2). For tri-repeats, the GAA type was most common, accounting for 7.17%, followed by the TTC repeat (5.87%), TCT repeat (4.58%), and AGA repeat (4.37%). The least frequent tri-repeats were ACT (0.14%) and TAG (0.17%). Among the longer repeat types, the 6-bp motif (29.62%) and 5-bp motif (28.59%) were more common, followed by the 7-bp motif (15.15%), 8-bp motif (9.51%), 9-bp motif (5.60%), 10-bp motif (3.22%), and other types (8.30%). Furthermore, we designed 4942 primers for 6711 identified SSR loci, including 2227 (45.06%) dinucleotide repeats, 2350 (47.55%) trinucleotide repeats, 94 (1.90%) tetranucleotide repeats, 11 (0.22%) pentanucleotide repeats, and 9 (0.18%) hexanucleotide repeats. Details of the SSR primers are summarized in Supplementary Table S3. 

### 2.6. Transcription Factors and WRKYs

**Figure 4:**
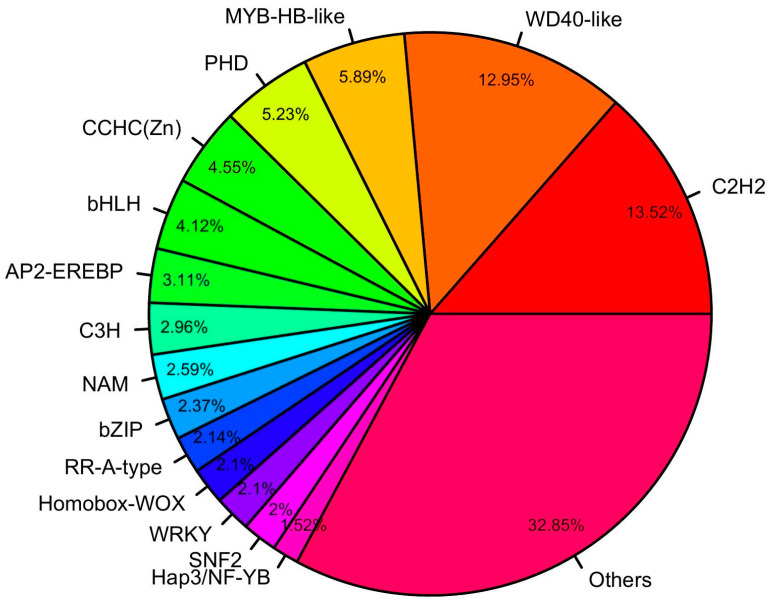
Transcription factors identified from the rice bean transcriptome.

Transcription factors are essential in various biological processes and regulate gene expression patterns. Therefore, we identified 4860 transcripts mapped to 98 unique families of transcription factors. Most of these belonged to the C2H2 (657) family, followed by WD40-like (629), MYB-HB-like (286), PHD (254), CCH (Zn) (221), and bHLH (200) (Figure 4). A total of 95 complete WRKY domain-containing unigenes were identified, and the amino acid length ranged from 103 to 747. We retrieved the 125 *Arabidopsis thaliana* WRKY proteins from the TAIR database and used them for phylogenetic analyses alongside rice bean WRKY proteins. Four major groups were formed: Group 1 contained the highest number of WRKY proteins, with 81 WRKY proteins, including 48 from Arabidopsis WRKY and 35 from rice bean.

**Figure 5:**
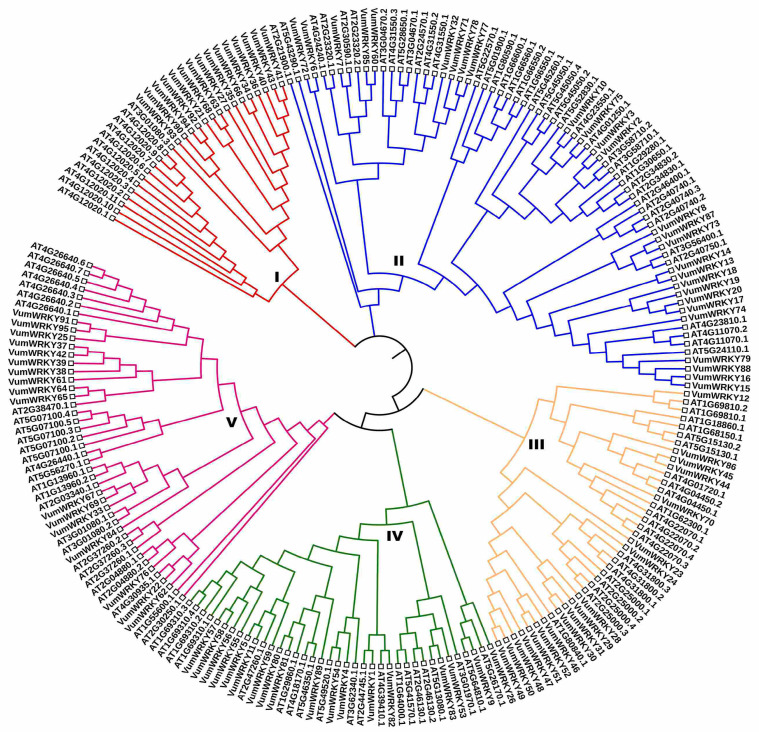
Phylogenetic analysis of WRKY transcription factors from rice bean and Arabidopsis.

Group IV had 70 WRKY proteins, including 27 from rice bean and 43 from Arabidopsis. Group III had the fewest WRKY proteins, with 27 in total, comprising 15 from rice bean and 12 from Arabidopsis (Figure 5).

### 2.7. VumWRKY Expression Pattern Under Aluminum Stress

**Figure 6:**
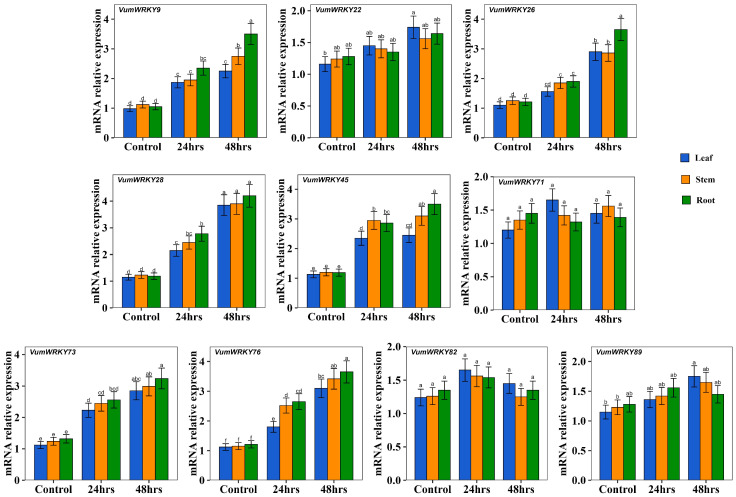
Expression of WRKY transcription factors analyzed in aluminum treated roots, stems, and leaves of rice bean by RT-qPCR analysis. The different letters indicate significant changes at *p* < 0.05.

Rice bean was treated with aluminum at different time points (0, 24 h, and 48 h; Supplementary Figure S1), and 10 WRKY transcription factors (*VumWRKY9*, *VumWRKY22*, *VumWRKY26*, *VumWRKY28*, *VumWRKY45*, *VumWRKY71*, *VumWRKY73*, *VumWRKY76*, *VumWRKY82*, and *VumWRKY89*) were selected based on their functional annotation and roles explained in other major crops (Supplementary Table S4). The expression pattern of these transcription factors were analyzed by RT-qPCR. Among the 10 WRKY transcription factors, six (*VumWRKY9*, *VumWRKY26*, *VumWRKY28*, *VumWRKY45*, *VumWRKY73*, and *VumWRKY76*) were highly expressed in roots, stems, and leaves at 24 h and 48 h, while the others *(VumWRKY22*, *VumWRKY71*, *VumWRKY82*, and *VumWRKY89)* did not show significant changes. The expression patterns of the WRKY transcription factors are presented in Figure 6.

## 3. Discussion

We generated the rice bean transcriptome assembly in this study, revealing 74,926 unigenes with a median length, N50, and GC content of 1138.24 bp, 1710 bp, and 40.99%, respectively. The median length and N50 sizes of the unigenes in this study were comparable to the previous studies reported in *Vigna* species, such as rice bean (median length = 986 bp, N50 = 1677 bp), mung bean (median length = 739 bp, N50 = 1176 bp), urd bean (median length = 1543 bp, N50 = 1928 bp), and cowpea (median length = 871 bp, N50 = 1534 bp) [26,27,28,29]. These findings suggest that the transcriptome data from our study were successfully assembled. A total of 59,850 unigenes (79.87%) out of 74,926 assembled unigenes were functionally annotated by searching against the Nr, Nt, UniProt, KEGG, Pfam, KOG, and GO databases. Upon conducting the similarity search, it was found that 58,470 unigenes could be annotated to the Nr database, whereas, 56,370 unigenes had similarity with UniProt, 49,667 with KOG, and 45,905 with the Pfam database.

The top-hit species distribution analysis revealed that most of the rice bean unigenes had significant homology with common bean (36.85%) sequences, followed by adzuki bean (34.12%) and mung bean (9.32%). This is due to the fact that the common bean, adzuki bean and mung bean all belong to the *Fabaceae* family. To enhance understanding of the role of these unigenes, additional classification was carried out using GO, KOG, and KEGG. A total of 15,593, 20,770, and 15,385 unigenes were found in the domains of BP, MF, and CC, respectively. KOG-mapped unigenes were categorized into 25 major groups. KEGG analysis mapped 18,081 unigenes into the top 20 major pathways. Most of the identified unigenes were mapped to the metabolic pathways, followed by biosynthesis of secondary metabolites, plant hormone signal transduction, plant–pathogen interaction, starch and sucrose metabolism, and MAPK signaling pathway. These findings will aid in understanding the critical roles that these genes play in the biosynthesis of active compounds in rice bean. Additionally, these findings will serve as a foundation for further research into the secondary metabolite pathways found in rice bean.

A total of 4860 transcription factors were identified, representing 98 unique transcription factor families, with the majority of the transcripts from the C2H2, WD40-like, MYB-HB like, and PHD families. Zinc finger proteins of the C2H2 type form a significant gene group involved in various aspects of plant development, particularly in the growth of roots and flowers, and play essential roles in responding to both biotic and abiotic stresses [30]. Proteins resembling WD40 are known to be pivotal players in multiple plant functions, including the intricate process of floral development, the transmission of light signals within the plant, the bolstering of innate immune responses, and the regulation of secondary metabolic pathways [31]. MYB transcription factors (TFs), constituting one of the most extensive gene families in plants, are pivotal in many biological processes, including plant growth and development, cellular morphology and patterning, metabolic activities, both primary and secondary metabolic pathways, and adaptation to environmental stresses [32]. Priyavathi et al. [33] reported the C2H2, WD40-like, CCHC (Zn), AP2EREBP, and WRKY transcription factors involved in various secondary metabolites pathways and responses to environmental stress.

Research on WRKY transcription factors, one of the largest families of transcription factors in plants, has advanced to the molecular mechanism level, garnering significant attention. Numerous WRKY transcription factor genes have been identified, and they play a major role in regulating abiotic stress response [34]. For instance, *OsWRKY22* enhances aluminum tolerance by activating *OsFRDL4* expression [35], and *AtWRKY47* confers aluminum tolerance by regulating cell wall-modifying genes [36]. *AtWRKY46* acts as a transcriptional repressor of *AtALMT1*, negatively regulating aluminum tolerance [37]. Despite this progress, there is a notable lack of research on the role of WRKY transcription factors in alleviating aluminum toxicity in rice bean, highlighting the need for further investigation in this area. In this study, we characterized the WRKY transcription factors from rice bean and studied their response to aluminum stress. Furthermore, we selected several transcription factors based on their functional annotation and roles identified in other major crops. Rice bean roots, stems, and leaves treated with aluminum at different time points (0, 24 h, and 48 h), and the expression pattern of 10 WRKY transcription factors were analyzed. Among the 10 WRKY transcription factors analyzed, six WRKYs—*VumWRKY9*, *VumWRKY26*, *VumWRKY28*, *VumWRKY45*, *VumWRKY73*, and *VumWRKY76*—were highly expressed at 24 h and 48 h, while the others did not show significant changes. Notably, the rice bean WRKY protein *VumWRKY45* had a close association with *AT4G01720.1* (WRKY 47) from Arabidopsis. Li et al. [36] reported that overexpression of the WRKY47 gene of Arabidopsis enhances aluminum tolerance by regulating cell wall-modifying genes like *ELP* and *XTH17* and balances aluminum distribution between the root apoplast and symplast. Similarly, the WRKY proteins *VumWRKY9* and *VumWRKY28* showed close structural associations with soybean WRKY proteins *GmWRKY21* (*NP_001237327.2*) and *GmWRKY81* (*Glyma.04G061400.1.p*), which have been reported to show aluminum tolerance [24,38]. The *GmWRKY21* transcription factor promotes tolerance by regulating major aluminum responsive genes like *ALMT*, *ALS3*, *MATE*, and *STOP1* and abiotic stress responsive genes *viz*., *KIN1*, *COR15A*, *COR15B*, *COR47*, *GLOS3*, and *RD29A* [24]. The *GmWRKY81* was overexpressed in an aluminum stress-tolerant soybean line, resulting in improvements in root architectural traits and higher proline levels and lower MDA levels under stress [24]. Among the studied WRKYs, three WRKYs—*VumWRKY9*, *VumWRKY28*, and *VumWRKY45*—are potential candidates for contributing to aluminum tolerance in rice bean. Although the current study identifies several WRKYs, it lacks functional evidence to confirm their role in conferring tolerance. Therefore, future research should employ functional genomics approaches, such as Virus-Induced Gene Silencing (VIGS) or genetic transformation, to validate the functions of these WRKYs and confirm their contributions to tolerance.

Further, we detected 6711 SSRs from 74,926 unigenes and designed 4942 SSR markers. A higher number of identified SSRs in a species indicates the cultivar’s strong adaptability to the environment [39]. Using previous transcriptome data, 3011 SSRs in rice bean, 13,134 in mung bean, and 1840 in urd bean were identified and further utilized [26,40,41]. Di-nucleotide repeats (52.53%) were abundant in our study, followed by tri-repeats (43.63%), tetra-repeats (2.89%), hexa-repeats (0.52%), and penta-repeats (0.43%). Our findings produced similar results to previous studies in terms of the abundance of di-repeats in cowpea [29] and pigeon pea [42]. However, studies on other legumes *viz.*, faba bean [43] and mung bean [40] showed the predominance of tri-repeats over other repeats. In the case of di-repeats, the AG-type repeat was more frequent than other types, which is in agreement with results for mung bean [29], barnyard millet [44], *Dalbergia odorifera* [45], and foxtail millet [46]. The GAA type of tri-repeats was more abundant in our study, which is similar to results for pigeon pea [42], Indian mulberry [47], and radish [48]. The newly developed SSRs have the potential to contribute significantly to upcoming research endeavors focused on exploring diversity, genetic linkage mapping, and the identification of functional genes in rice bean and its closely related species.

## 4. Materials and Methods

### 4.1. Plant Genetic Materials and RNA Isolation

The genetically pure rice bean accession “TNAU RED” was obtained from the Agricultural College and Research Institute, Tamil Nadu Agricultural University, Eachangkottai, India. RNA was isolated from the leaves using the RNeasy plant mini kit (Qiagen, Hilden, Germany). The quantity and quality of RNA were then measured using a biospectrometer (Eppendorf, Hamburg, Germany). The RNA integrity value (RIN) (>7.0) was confirmed by the Agilent Bioanalyzer 2100 system (Agilent Technologies, Santa Clara, CA, USA).

### 4.2. Library Preparation and High-Throughput Sequencing

Following the quality check of the RNA samples, the samples were used to construct the library with the support of the Illumina TruSeq^™^ RNA sample preparation kit, following the manufacturer’s recommendations (Illumina Inc., SanDiego, CA, USA). After confirming the library quality, sequencing was conducted by the Illumina HiSeq 2000 platform with paired-end (PE) reads of 101 bp. The raw reads were submitted to the NCBI SRA (http://www.ncbi.nlm.nih.gov/Traces/sra) under Bio Project accession number PRJNA900023.

### 4.3. Data Filtering and De Novo Assembly

The paired-end raw reads were tested using FastQC v0.12.1 to confirm the per base quality and adapter contamination [49]. The Trimmomatic v0.39 was used for quality filtering and the removal of adapter sequences with the following considerations: ILLUMINACLIP:TruSeq3-PE-2.fa:2:30:10 AVGQUAL:20 LEADING:20 TRAILING:20 MINLEN:30 [50]. The resulting high-quality paired-end reads were de novo assembled into transcripts (>300 bp) using Trinity (v2.15.1). To obtain non-redundant transcripts, the assembled transcripts were clustered using CD-HIT EST v4.8.1 at 90% identity and 95% query coverage.

### 4.4. Functional Annotation and Classification

For functional annotation, the assembled unigenes were subjected to BLASTx homology search against seven databases: the NCBI non-redundant protein database (Nr), NCBI non-redundant nucleotide database (NT), UniProt-Viridiplantae database, Protein family (Pfam), KEGG Ortholog database, Gene Ontology (GO) database, and Clusters of Orthologous Groups of proteins (KOG) database, with an E-value cut-off ≤ 1 × 10^−5^. The resulting BLAST xml file was imported into OmicsBox v2.0.36 for assignment to various GO terms describing cellular components, biological processes, and molecular function categories. The KOBAS server was used to assign the KO IDs to the unigenes, and the KEGG mapper was used to assign pathways to the annotated unigenes (https://www.genome.jp/kegg/mapper/ accessed on 7 July 2023). 

### 4.5. Identifcation of Transcription Factors and WRKY

The TransDecoder v5.7.1 was used to predict the longest ORFs in the transcripts [51]. The plantTFcat analysis tools (https://www.zhaolab.org/PlantTFcat/ accessed on 10 July 2023 were used to predict transcription factors in protein sequences by InterProScan domain pattern searches, ensuring the high coverage and sensitivity of genome-scale sequences [52]. The HMMER v3.1 was employed for Hmmscan of protein sequences against the Pfam database to predict the WRKY family containing unigenes. Additionally, an NCBI CDD search and the ExPASy–PROSITE tool were used to ensure the presence of conserved WRKY domains, and unigenes with short and incomplete WRKY domains were removed. The WRKY proteins were further aligned using the multiple sequence alignment tool ClustalW (http://www.ebi.ac.uk/clustalw/ accessed on 12 July 2023), employing default parameters. The aligned sequences were then used for phylogenetic tree construction using the neighbor-joining method in MEGA 11 software [53].

### 4.6. Detection of SSRs

Simple sequence repeats were detected using the MIcroSAtellite identification tool v1.0 (MISA) available at (http://pgrc.ipk-gatersleben.de/misa/ accessed on 5 August 2023). A unit size threshold of 6 was applied to identify di-nucleotide repeats, while a threshold of 5 was used for SSRs with 3-, 4-, 5-, and 6-nucleotide repeats. Primer pairs for SSR markers were designed by using sequences bordering the designated microsatellite loci with the support of the Premier3 software package. The PCR product sizes spanned from 100 to 300 base pairs [54].

### 4.7. Aluminum Stress and RT-qPCR Analysis

Rice bean seeds were surface sterilized with 75% ethanol for 2 min, followed by 5% sodium hypochlorite for 5 min, and rinsed with sterile water. The seeds were sown on roll towels soaked in 100 µM CaCl_2_ (pH 7.0) and kept in the dark at 25 °C with proper aeration. After 7 days, uniform seedlings were transferred to a hydroponic system containing half-strength Hoagland nutrient solution (pH 4.5) for 3 days [55]. Aluminum stress was applied by adding 100 µM AlCl_3_·6H_2_O to the nutrient solution. A separate control group without aluminum stress was maintained. The experiment followed a completely randomized block design with three replications, each consisting of 10 plants per replication. The nutrient solution was replaced daily to maintain consistent aluminum concentrations and pH levels. Root, stem, and leaf samples were collected at 0, 24, and 48 h; frozen in liquid nitrogen; and stored at −80 °C for further analysis. Total RNA was isolated using the RNeasy plant mini kit and converted to cDNA using the iScript cDNA synthesis kit following the product guidelines (Bio-Rad Laboratories, Inc., Hercules, CA, USA). RT-qPCR analysis was performed using the Bio-Rad CFX 96 (Bio-Rad Laboratories, Inc., Hercules, CA, USA). The 20 μL reaction mixture consisted of 1 μL cDNA, 1 μL forward and reverse primers, 10 μL AccuPower 2X Greenstar SYBR Green Master mix, and 6 μL sterile H_2_O. Target gene expression was analyzed using the 2^−ΔΔCt^ method, with Actin as the internal control (Supplementary Table S5). All samples were amplified in three biological replicates, with three technical replicates each.

## 5. Conclusions

In summary, we generated a comprehensive transcriptome dataset for rice bean and performed de novo assembly, followed by functional annotation of the unigenes. Additionally, we developed a set of SSR markers and characterized the response of WRKY genes to aluminum stress in rice bean. Collectively, the results obtained from this study extend a genomic resource for rice bean and provide novel insights for further research on the role of WRKY transcription factors in aluminum tolerance in rice bean.

## Figures and Tables

**Table 1:** Raw reads and clean reads details of *Vigna umbellata* transcriptome.

S. No.	Samples	Raw Reads	Clean Reads	Read Length	Clean Bases	GC (%)	Error (%)	Q20 (%)	Q30 (%)
1	R_1_	25,393,114	25,161,907	101	2.54 GB	43	0.036	98.50	95.55
2	R_2_	21,173,765	20,982,282	101	2.12 GB	43	0.035	98.75	94.85

**Table 2:** Details of transcript length distribution and unigenes assembled.

S. No.	Characteristics	Transcript	Unigenes
1	Contig N10	4221	4084
2	Contig N20	3208	3089
3	Contig N30	2610	2501
4	Contig N40	2164	2070
5	Contig N50	1801	1710
6	Contig N90	516	474
7	300–500 bp	26,857	24,764
8	500–1000 bp	25,077	21,007
9	1000–2000 bp	23,250	17,608
10	>2000 bp	15,749	11,547
11	Average length	1216.89	1138.24
12	Minimum length	301	301
13	Maximum length	24,052	24,052
14	Total Transcripts	90,933	74,926
15	Total assembled bases	110,655,186	85,283,755
16	GC (%)	41.01	40.99

**Table 3:** Functional annotations of unigenes against seven databases.

S. No.	Annotated Database	Numbers	Percentage (%)
1	Total unigenes	74,926	
2	Nr annotated	58,470	78.03
3	Nt annotated	55,350	73.87
4	UniProt annotated	56,370	75.23
5	KEGG annotated	28,378	37.87
6	Pfam	45,905	61.27
7	KOG annotated	49,667	66.29
8	GO annotated	27,095	36.16
9	At least one	59,850	79.87

**Table 4:** Summary of SSR types identified in rice bean transcriptome.

Items	Number
**SSR Search Results**	
Total number of sequences examined	74,926
Total size of examined sequences (bp)	85,283,755
Total number of identified SSRs	6711
Number of SSRs containing sequences	5700
Number of sequences containing more than 1 SSR	840
Number of SSRs present in compound formation	472
**Distribution to different repeat-type classes**	
Di-nucleotides	3525
Tri-nucleotides	2928
Tetra-nucleotides	194
Penta-nucleotides	29
Hexa-nucleotides	35

## Data Availability

The data presented in this study are available within the article. All fastq files have been submitted to the NCBI Sequence Read Archive database at https://www.ncbi.nlm.nih.gov/sra. The NCBI accession for this project is PRJNA900023.

## Supplementary Materials

The following supporting information can be downloaded at: https://www.mdpi.com/article/10.3390/plants13223170/s1, Figure S1: Rice bean plants response to aluminum stress; Table S1: Description of KOG mapped transcripts with their corresponding enzyme codes; Table S2: Frequency of identified SSR; Table S3: The designed SSR primer details; Table S4: Summary of selected WRKY transcription factors for aluminum stress response studies; Table S5: The qRT PCR primers used in this study. References [56,57,58,59,60,61,62] are cited in the supplementary materials.
