# Supplementary figures and images for: De Novo Transcriptome Assembly of Rice Bean (Vigna umbellata) and Characterization of WRKY Transcription Factors Response to Aluminum Stress
Source: Plants (Basel). 2024 Nov 12;13(22):3170. doi: 10.3390/plants13223170 (PMC11598158; doi:10.3390/plants13223170)

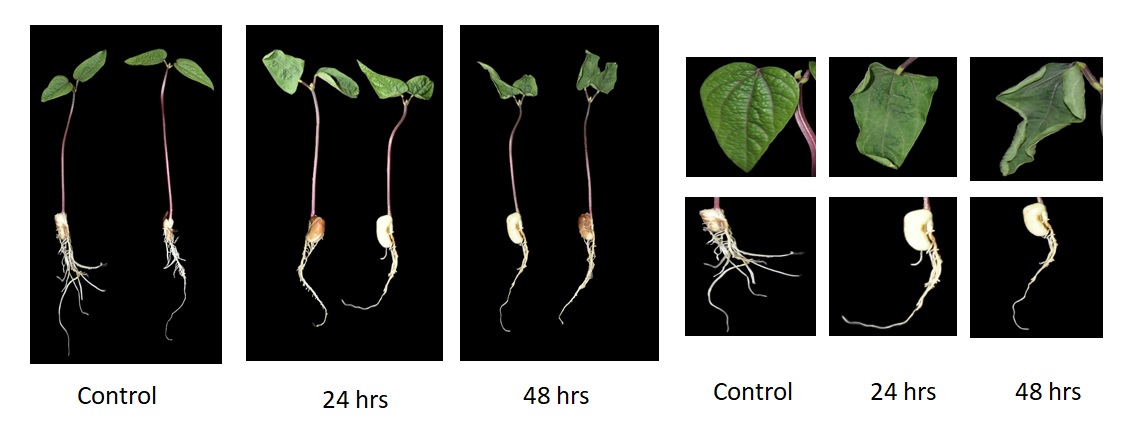


**Supplementary figure 1**. Rice bean plants response to aluminum stress

Supplement: Supplementary file 1 [file plants-13-03170-s001.zip › 1. Supplementary figures.docx]
